# The transcription factor CREB acts as an important regulator mediating oxidative stress-induced apoptosis by suppressing αB-crystallin expression

**DOI:** 10.18632/aging.103474

**Published:** 2020-06-17

**Authors:** Ling Wang, Qian Nie, Meng Gao, Lan Yang, Jia-Wen Xiang, Yuan Xiao, Fang-Yuan Liu, Xiao-Dong Gong, Jia-Ling Fu, Yan Wang, Quan Dong Nguyen, Yizhi Liu, Mugen Liu, David Wan-Cheng Li

**Affiliations:** 1 The State Key Laboratory of Ophthalmology, Zhongshan Ophthalmic Center, Sun Yat-Sen University, Guangzhou 510230, Guangdong, China; 2 Key Laboratory of Molecular Biophysics of Ministry of Education, College of Life Science and Technology, Center for Human Genome Research, Huazhong University of Science and Technology, Wuhan 430074, Hubei, China; 3 Medical College, Henan University of Science and Technology, Luoyang 471000, Henan, China; 4 Byers Eye Institute, Stanford University School of Medicine, Palo Alto, CA 94303, USA

**Keywords:** CREB, αB-Crystallin, oxidative stress, gene regulation, apoptosis, lens

## Abstract

The general transcription factor, CREB has been shown to play an essential role in promoting cell proliferation, neuronal survival and synaptic plasticity in the nervous system. However, its function in stress response remains to be elusive. In the present study, we demonstrated that CREB plays a major role in mediating stress response. In both rat lens organ culture and mouse lens epithelial cells (MLECs), CREB promotes oxidative stress-induced apoptosis. To confirm that CREB is a major player mediating the above stress response, we established stable lines of MLECs stably expressing CREB and found that they are also very sensitive to oxidative stress-induced apoptosis. To define the underlying mechanism, RNAseq analysis was conducted. It was found that CREB significantly suppressed expression of the αB-crystallin gene to sensitize CREB-expressing cells undergoing oxidative stress-induced apoptosis. CREB knockdown via CRISPR/CAS9 technology led to upregulation of αB-crystallin and enhanced resistance against oxidative stress-induced apoptosis. Moreover, overexpression of exogenous human αB-crystallin can restore the resistance against oxidative stress-induced apoptosis. Finally, we provided first evidence that CREB directly regulates αB-crystallin gene. Together, our results demonstrate that CREB is an important transcription factor mediating stress response, and it promotes oxidative stress-induced apoptosis by suppressing αB-crystallin expression.

## INTRODUCTION

Aging refers to the progressive loss of tissue and organ functions over time. It is well established that reactive oxygen species (ROS) derived from action of various oxidases such as nicotinamide adenine dinucleotide phosphate (NADPH) oxidase and lipoxygenase can cause damages to DNA, proteins and membrane lipids [1]. The accumulation of the oxidative stress-induced damages in these different macromolecules causes age-associated functional loss in different tissues and organs [2], accounting for the core of the oxidative stress theory of aging. The cellular ROS components include superoxide anion (O_2_·), hydroxyl ion (OH·) and hydrogen peroxide (H_2_O_2_) [1]. Although H_2_O_2_ is not a free radical, through the Fenton or Haber-Weiss reaction, it can generate hydroxyl radicals which are extremely reactive, causing damage to proteins in cytoplasm and phospholipids in cellular membrane [1–2]. In the human eye, it has been reported that the level of H_2_O_2_ is elevated in the aqueous humor from less than 25 μM in normal lens to more than 50 μM in cataract patients [3]. Oxidative stress has been considered as one of the initiating factors in the formation of cataract, an essential aging disease that causes blindness in developing countries [4].

The cAMP response element binding protein, CREB is a general transcription factor, and its most prominent function has been shown to mediate synaptic plasticity associated with long-term memory [5–30]. Disruption of CREB in mice causes defects in long-term potentiation and long-term memory [19]. On the other hand, expression of the dominant-active CREB polypeptide accelerates the learning process [20, 21]. The CREB control of synaptic plasticity occurs through its regulation of a panel of genes implicated in synthesis of neuropeptides and neurotransmitters [12–15, 17–19, 22–24].

CREB also promotes growth factor-dependent survival of both sympathetic and cerebellar neurons [13, 25–27]. Nerve growth factor (NGF) and brain-derived neurotrophic factor (BDNF) have been shown to enhance survival of the above types of neurons [13, 25–27]. At the molecular level, it has been shown that NGF and BDNF activate the RSK90 kinase, which phosphorylates CREB at S133 to promote expression of the anti-apoptotic gene Bcl-2 [28].

In addition, knockout study reveals that CREB regulates cell proliferation. The CREB (-/-) mice die at birth with impaired T-cell development [29]. Mice with expression of S133A mutation develop dwarfism due to somatotroph hypoplasia, which is due in part to a block in cell proliferation [30].

Although CREB functions in mediating synaptic plasticity associated with long-term memory, growth factor-dependent cell proliferation and survival have been well established [10–13, 15–22, 26–28], its function mediating stress response remains elusive.

Cataract is an aging disease that in most cases is derived from aging process or stress induction such as oxidative stress [4 and references therein]. Mechanistically, we have previously demonstrated that stress-induced apoptosis is a common cellular basis for non-congenital cataractogenesis [31–32].

αB-Crystallin is a major lens protein that has a structural role in maintaining the transparency of the lens [33, 34]. It is a member of the small heat shock protein (HSP) family [35]. αB-crystallin is mainly expressed in the ocular lens. In addition, it is also expressed outside of the lens in a number of tissues such as skeletal and cardiac muscles and to a lesser extent in skin, brain, and kidney [36–38]. Besides its structural role, αB-crystallin has been shown to act as molecular chaperone [36–51], autokinase [52], and antiapoptotic regulators [53–75]. Although the protective role of αB-crystallin against stress conditions such as oxidative stress has been well documented [76–81], its regulation by upstream factors remains to be further characterized.

In the present study, we first determined that rat lens organ culture treated by oxidative stress underwent apoptosis, and associated with the apoptotic process, we observed that CREB was transiently upregulated, and in contrast, αB-crystallin expression was downregulated. Cells expressing wild type CREB was very sensitive to hydrogen peroxide-induced apoptosis. To define the underlying mechanism, we have conducted RNAseq analysis and subsequent confirmation studies. Our results revealed that CREB completely suppresses expression of the αB-crystallin gene in mouse lens epithelial cells to sensitize the CREB-expressing cells undergoing stress-induced apoptosis. Knockdown of CREB via CRISPR/CAS9 technology led to upregulation of αB-crystallin and enhanced resistance against oxidative stress-induced apoptosis. In addition, overexpression of exogenous human αB-crystallin can also inhibit the stress-induced apoptosis to a large degree in CREB-expressing cells, indicating that CREB-mediated suppression of αB-crystallin gene is a major mechanism for its promotion of stress-induced apoptosis. Finally, using EMSA and ChIP assays, for the first time, we demonstrated that CREB directly regulates αB-crystallin gene by binding to upstream and downstream enhancer elements. Together, our results demonstrate that CREB is an important transcription factor regulating stress response, and it does so by suppressing αB-crystallin expression.

## RESULTS

### Treatment of rat lens organ culture with oxidative stress induces apoptosis of lens epithelial cells, which is linked to down-regulation of αB-crystallin but up-regulation of CREB

**Figure 1:**
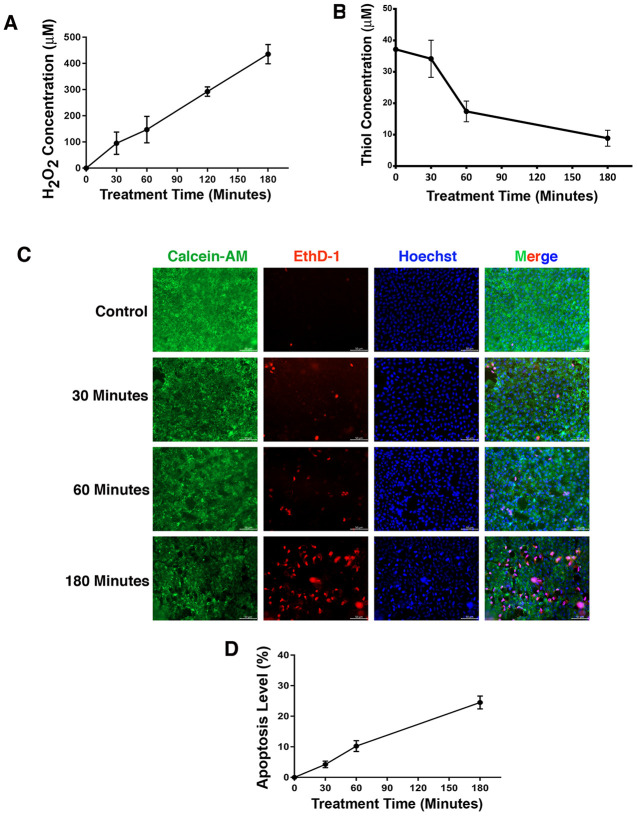
**Treatment of rat lens with 40 mU GO caused apoptosis of lens epithelial cells.** (**A**) Dynamic H_2_O_2_ concentration generated from 40mU glucose oxidase (GO) in the M199 medium in which rat lenses were cultured in 10-cm culture dish with 30 ml medium. (**B**) Dynamic changes of free thiol levels in rat lens epithelial cells under 40 mU GO treatment. (**C**) Live/dead assays to reveal time-dependent apoptosis of rat lens epithelial cells under treatment of 40 mU GO. Green fluorescence represents live cells as detected by Calcein-AM, and red fluorescence detected by EthD-1 refers to dead cells. (**D**) Apoptotic rate of rat lens epithelial cells under 40 mU GO treatment. All experiments were repeated three times. Error bar represents standard deviation, N=3.

**Figure 2:**
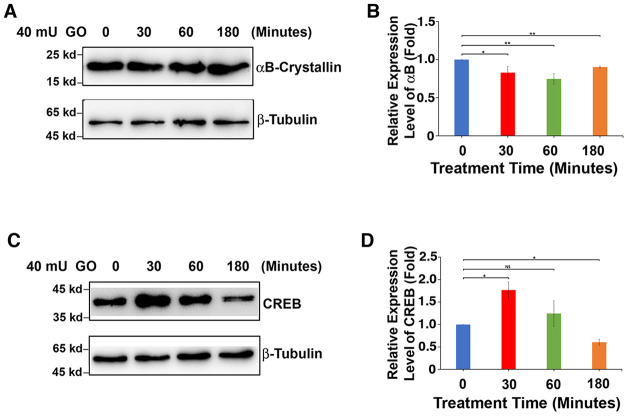
**GO-induced apoptosis of rat lens epithelial cells is derived from downregulated expression of αB-crystallin caused by upregulation of CREB.** (**A**) Western blot analysis of αB-crystallin in rat lens epithelium with 40mU glucose oxidase treated from 0 to 180 minutes. (**B**) Semi-quantification of the western blot results in (**A**). (**C**) Western blot analysis of total CREB (T-CREB) in rat lens epithelium with 40mU glucose oxidase treated from 0 to 180 minutes. (**D**) Semi-quantification of the western blot results in (**C**). Note the reverse relationship between expression of αB-crystallin with that of total CREB expression. All experiments were repeated three times. Error bar represents standard deviation, N=3. * p<0.05; ** p<0.01; NS, statistically not significant.

It is well established that oxidative stress has an initiating role in cataractogenesis [4, 31–32]. In deducing the underlying cellular mechanism, we have previously demonstrated that oxidative stress first induces apoptosis of lens epithelial cells followed by subsequent cataractogenesis [31, 32]. To further explore how oxidative stress causes apoptosis of lens epithelial cells, we treated rat lenses with 40 mU glucose oxidase (GO) for 0 to 3 hours. Consistent with our previous studies [31–32], GO treatment generated hydrogen peroxide (Figure 1A) and caused significant drop of the free thiol level (Figure 1B). As a result, the epithelial cells of the treated rat lens were induced to undergo apoptosis (Figure 1C and 1D). More importantly, we observed that GO treatment caused significant downregulation of αB-crystallin expression in 30 minutes (Figure 2A, 2B). Paralleling to downregulation of αB-crystallin expression, GO induced transient upregulation of CREB expression in 30 minutes (Figure 2C, 2D). Subsequently, as CREB expression became attenuated, expression of αB-crystallin appeared slightly restored in 180 minutes, suggesting that CREB seemed to negatively regulate αB-crystallin to promote apoptosis (Figure 2B, 2D).

### Mouse lens epithelial cells expressing CREB are more sensitive to oxidative stress-induced apoptosis

**Figure 3:**
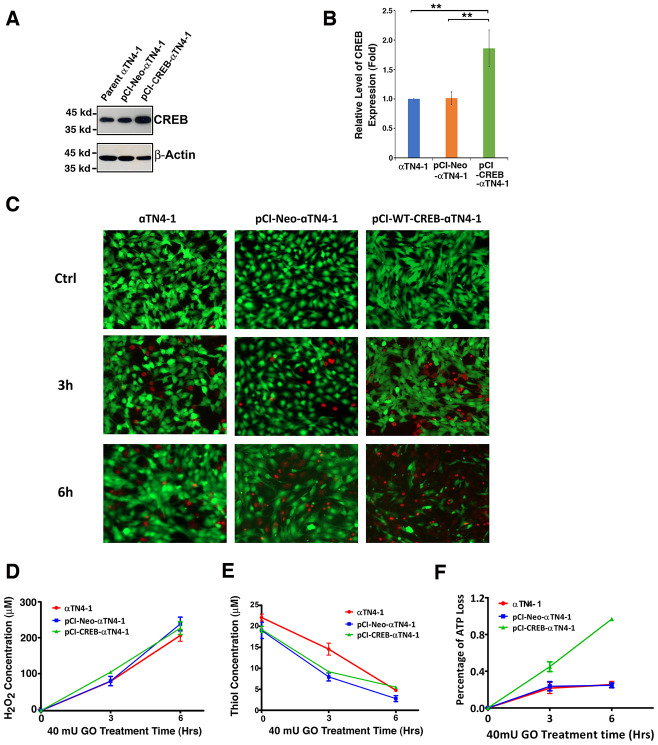
**The expression of exogenous CREB sensitizes mouse lens epithelial cells to 40 mU GO-induced apoptosis (C, F).** (**A**) Western blot analysis of the CREB levels in αTN4-1, pCI-αTN4-1, and pCI-CREB-αTN4-1 cells. (**B**) Semi-quantification of the western blot results in (**A**). (**C** and **F**) The αTN4-1, pCI-αTN4-1, and pCI-CREB-αTN4-1 cells were grown to 90% confluence. Then, 40 mU GO was added into the 3 types of cells, and the 3 types of cells were treated for indicated time. At the end of treatment, the cells were harvested for either live/dead assays (**C**), or for CellTiter-Glo® Luminescent Cell Viability Assay analysis [89] (**F**) to determine the rate of apoptosis. Note that pCI-CREB-αTN4-1 cells displayed the highest level of apoptosis (nearly 100%) in the 40mU glucose oxidase treatment (**F**). Green fluorescence represents live cells as detected by Calcein-AM, and red fluorescence detected by EthD-1 refers to dead cells. (**D**) Dynamic H_2_O_2_ concentration generated from 40mU glucose oxidase (GO) in the DMEM medium. (**E**) Dynamic changes of free thiol levels in mouse lens epithelial cells cultured in the DMEM medium under 40 mU GO treatment. All experiments were repeated three times. Error bar represents standard deviation, N=3. ** p<0.01.

To test if CREB could suppress αB-crystallin expression to promote oxidative stress-induced apoptosis, we first established stable lines of lens epithelial cells expressing the empty vector, pCI-αTN4-1, or wild type CREB, pCI-CREB-αTN4-1. Expression of exogenous wild type CREB was determined using western blot analysis and immunofluorescence. As show in Figure 3A and 3B, wild type CREB was clearly overexpressed. Both endogenous and exogenous CREB were localized in the nuclei (Supplementary Figure 1).

Next, we treated different lines of lens epithelial cells, αTN4-1, pCI-αTN4-1 and pCI-CREB-αTN4-1 with 40 mU glucose oxidase (GO) for 6 hours (Figure 3C and 3F). Hydrogen peroxide was consistently generated from 3 to 6 hours (Figure 3D). At the same time, the free thiol levels in these cells were significantly downregulated (Figure 3E). Live/dead viability/cytotoxicity assay and ATP loss analysis [89] revealed that cells expressing wild type CREB were most sensitive to GO-induced apoptosis (Figure 3C and 3F). A 6-hour treatment with 40 mU GO caused ATP loss in more than 90% cells expressing wild type CREB (Figure 3F). Thus, our results revealed that expression of exogenous wild type CREB sensitizes lens epithelial cells to oxidative stress-induced apoptosis.

### RNAseq analysis revealed that expression of exogenous CREB significantly downregulates αB-crystallin gene in lens epithelial cells

**Figure 4:**
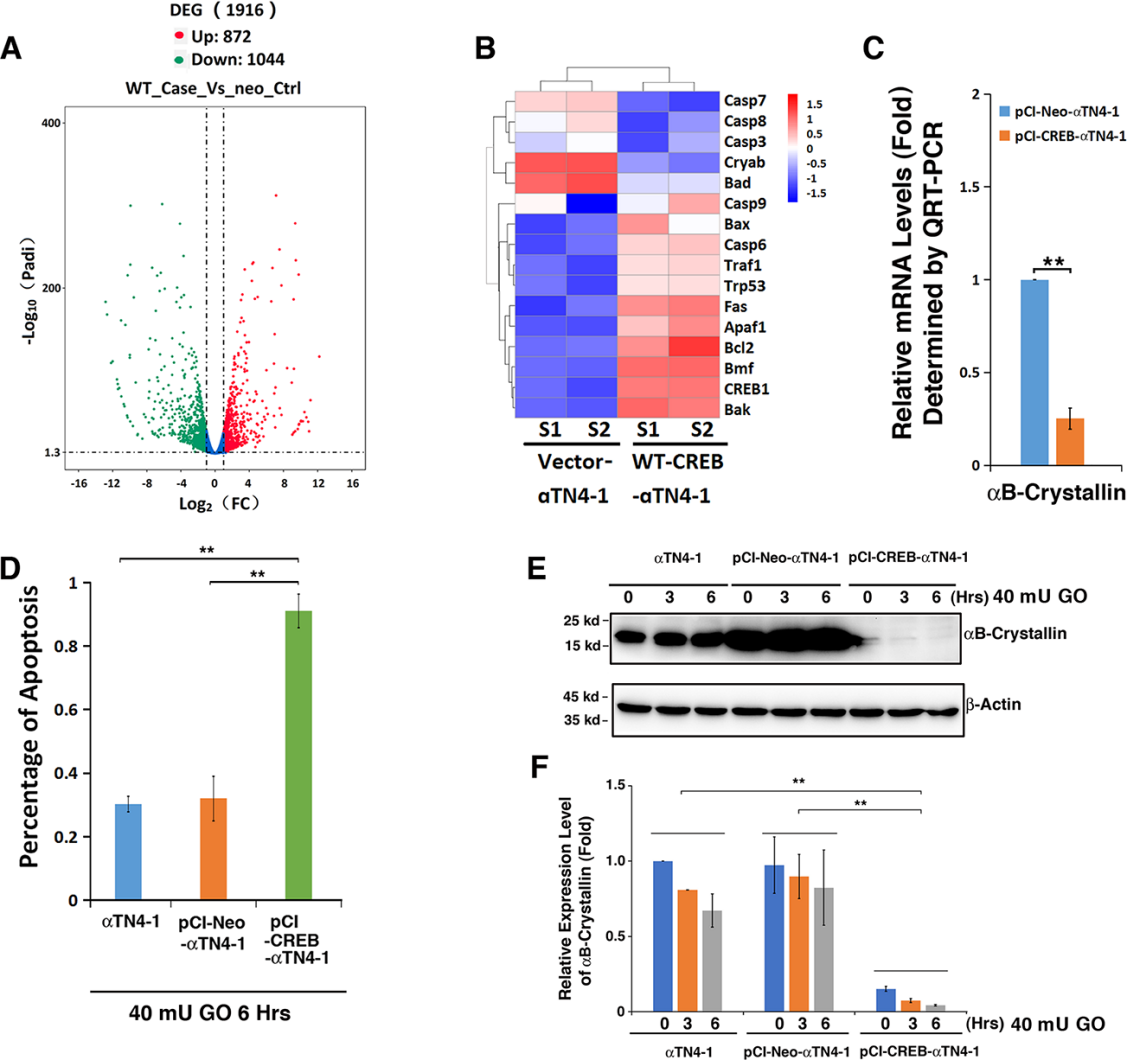
**Comparative transcriptome analysis.** (**A**–**C**) Both pCI-αTN4-1 and pCI-CREB-αTN4-1 cells were grown to 90% confluence and then harvested for RNAseq analysis. The gene expression patterns between vector-transfected cells and wild type CREB-transfected cells were compared (SRA accession: PRJNA566306). Compared to pCI-vector, expression of the exogenous WT-CREB caused changes in the expression patterns of 1916 genes, 872 genes were upregulated and 1044 genes were downregulated (**A**). (**B**) Hierarchical cluster analysis of apoptosis-associated genes. (**C**) The expression levels of the anti-apoptotic gene αB-crystallin in pCI-αTN4-1 and pCI-CREB-αTN4-1 cells (**B**) were further verified by qRT-PCR. Note that the expression of anti-apoptotic gene coding for αB-crystallin was significantly downregulated in pCI-CREB-αTN4-1 cell. (**D**–**F**) CREB downregulates expression of αB-crystallin during H_2_O_2_-induced apoptosis of CREB-expressing cells. (**D**) Apoptosis rate changes in αTN4-1, pCI-αTN4-1 and pCI-CREB-αTN4-1 cells under treatment of 40 mU GO from 0 to 6 hours were measured by CellTiter-Glo® Luminescent Cell Viability Assay analysis [89]. (**E**) Western blot analysis of the expression levels of αB-crystallin in αTN4-1, pCI-αTN4-1 and pCI-CREB-αTN4-1 cells under 40 mU GO. Note that the expression level of αB-crystallin was significantly downregulated in pCI-CREB-αTN4-1 cell. In addition, 40 mU GO treatment downregulated expression level of αB-crystallin in αTN4-1, pCI-αTN4-1 and pCI-CREB-αTN4-1 cells. (**F**) Semi-quantification of the western blot results in E. All experiments were repeated three times. Error bar represents standard deviation, N=3. ** p<0.01.

To understand why CREB-expressing cells displayed strong sensitivity to oxidative stress insult, we conducted RNAseq analysis between wild type (WT) CREB transfected cell and vector-transfected cells. As shown in Figure 4A (SRA accession: PRJNA566306), overexpression of WT CREB altered expression patterns of 1916 genes among which 872 were upregulated, and 1044 were downregulated. These genes belong to various signaling pathways (Supplementary Figure 2). Among these genes, we noticed that the most striking gene with consistently changed expression pattern is the one coding for αB-crystallin (Figure 4B, Cryab). QRT-PCR analysis confirmed the RNAseq result about αB-crystallin expression in vector and CREB-expressing cells (Figure 4C). Thus, our results revealed that expression of exogenous CREB suppresses αB-crystallin expression to confer its hypersensitivity to stress response.

### Overexpression of CREB in mouse lens epithelial cells dramatically down-regulates endogenous αB-crystallin

To confirm the RNAseq analysis data and demonstrate that the αB-crystallin downregulation by CREB indeed accounts for the hypersensitivity of pCI-CREB-αTN4-1 cells to stress-induced apoptosis (Figure 4D), we conducted western blot analysis and examined the relative levels of αB-crystallin in all 3 types of cells under treatment by 40 mU GO for 3 or 6 hours (Figure 4E and 4F). As shown in Figure 4D, cells expressing CREB displayed quick ATP loss under 40 mU GO treatment. Consistent with quick loss of ATP, the expression of αB-crystallin in cells expressing CREB remained constantly very lower level, and 40 mU GO treatment further downregulated it to barely detectable level (Figure 4E and 4F). In αTN4-1 and pCI-αTN4-1cells, 40 mU GO also down-regulated the expression of αB-crystallin to certain degree. Thus, in CREB-expressing cells, loss of cell viability is closely linked to the suppression of αB-crystallin expression by CREB.

### Knockdown of endogenous CREB in lens epithelial cells upregulates αB-crystallin expression and confers resistance to oxidative stress-induced apoptosis

**Figure 5:**
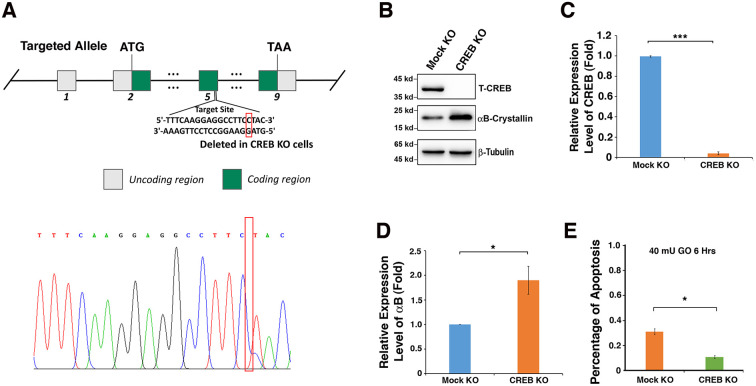
**Silence of CREB activates expression of αB-crystallin in αTN4-1 cells.** (**A**). CREB knockout strategy in αTN4-1 cells. The homozygous point deletion was created by CRISPR/CAS9 technology. A single nucleotide was deleted in exon 5, and the deletion mutation was verified by DNA sequencing. (**B**) Western blot analysis of the expression levels of CREB and αB-crystallin in αTN4-1 mock knockdown and CREB knockdown cells. (**C**, **D**) Semi-quantification of the western blot results in (**B**). (**E**) Apoptosis rate in, mock KO and αTN4-1 CREB KO cells under treatment of 40 mU GO for 6 hours measured by CellTiter-Glo® Luminescent Cell Viability Assay analysis [89]. All experiments were repeated three times. Error bar represents standard deviation, N=3. * p<0.05, *** p<0.005.

To confirm that CREB suppresses αB-crystallin expression, which affects the sensitivity of mouse lens epithelial cells to stress response, we used CRISPR/CAS9 technology to knockout expression of CREB in αTN4-1 cell (Figure 5A). The deletion of a single nucleotide in exon 5 was confirmed with DNA sequencing (Figure 5A) and the absence of CREB protein expression was verified by western blot analysis (Figure 5B, 5C). When CREB expression was significantly knocked down, the expression level of αB-crystallin gene was distinctly upregulated (Figure 5B–5D). Next, we treated Mock-KO-αTN4-1 and CREB-KO-αTN4-1 cells with 40 mU GO, and the cell viability was measured by ATP loss. As shown in Figure 5E, cells with knockdown of endogenous CREB expression and upregulation of endogenous αB-crystallin expression displayed much stronger resistance to oxidative stress-induced apoptosis than mock knockdown cells. Together, these results confirm that CREB is a negative regulator of αB-crystallin gene and it promotes oxidative stress-induced apoptosis of mouse lens epithelial cell by suppressing αB-crystallin expression.

### Overexpression of exogenous human αB-crystallin partially restores the resistance against stress-induced apoptosis of mouse lens epithelial cells expressing CREB

**Figure 6:**
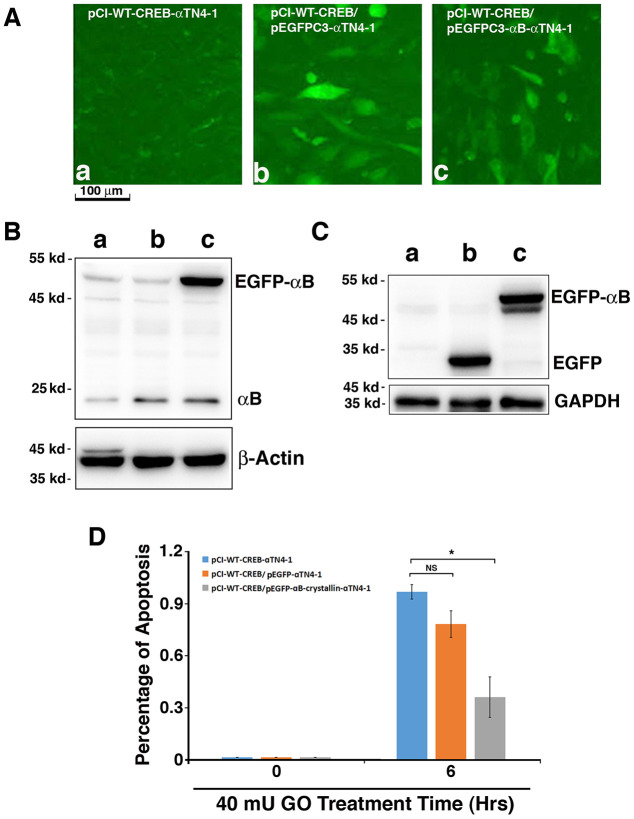
**Exogenous human αB-crystallin restores the ability of pCI-CREB-αTN4-1 cells against hydrogen peroxide-induced apoptosis.** (**A**) The pCI-CREB-αTN4-1 cells were either untransfected (A-a), or transfected with pEGFPC3 vector (A-b), or pEGFPC3-HαB (A-c) transiently. Transfection was confirmed by fluorescence microscopy. The pEGFPC3 vector-transfected pCI-CREB-αTN4-1 cells displayed homogenous distribution of green fluorescence protein in the whole cells (A-b). In contrast, in the pEGFPC3-HαB-transfected pCI-CREB-αTN4-1 cells, the green fluorescence fusion protein was largely restricted in the cytoplasm (A-c). (**B**) Western blot analysis of the expression level of endogenous αB-crystallin and GFP-αB fusion protein in pCI-CREB-αTN4-1 cells (a), pCI-CREB-αTN4-1/pEGFPC3-αTN4-1 cells (b) and pCI-CREB-αTN4-1/pEGFPC3-HαB-αTN4-1 cells (c) detected with anti-αB antibody. (**C**) Western blot analysis of the expression level of GFP and GFP-αB fusion protein in pCI-CREB-αTN4-1 cells (a), pCI-CREB-αTN4-1/pEGFPC3-αTN4-1 cells (b) and pCI-CREB-αTN4-1/pEGFPC3-HαB-αTN4-1 cells (c) detected with anti-EGFP antibody. (**D**) After treatment by 40 mU GO for 6 hours, apoptosis in the 3 types of cells as indicated were analyzed. Note that pCI-CREB-αTN4-1/pEGFPC3-HαB-αTN4-1 cells expressing exogenous HαB displayed over 50% less apoptosis than pCI-CREB-αTN4-1 cells and pCI-CREB-αTN4-1/pEGFPC3-αTN4-1 cells. All experiments were repeated three times. Error bar represents standard deviation, N=3. * p<0.05; NS, statistically not significant.

To further confirm that CREB-induced down-regulation of αB-crystallin was indeed the main reason for the enhanced apoptosis of the CREB-transfected cells under GO treatment, we next overexpressed human αB-crystallin (HαB) cDNA in pCI-CREB-αTN4-1 cells using pEGFPC3-HαB with the vector pEGFPC3 as control. Expression of EGFP or EGFP-HαB fusion protein can be distinguished by their localization (Figure 6A-b/c). While EGFP alone was homogenously expressed within the cells (Figure 6A-b), expression of EGFP-HαB was largely restrained in the cytoplasm (Figure 6A-c). The expression of exogenous EGFP and the fusion protein, EGFP-αB-crystallin were further verified by western blot analysis using antibodies against αB-crystallin (Figure 6B) and GFP (Figure 6C). Next, we compared the sensitivity of the 3 types of cells to 40 mU GO-induced apoptosis. As shown in Figure 6D, expression of human αB-crystallin cDNA in pCI-CREB-αTN4-1 cells decreased more than 50% of 40 mU GO-induced apoptosis. Together, these results further demonstrated that CREB sensitizes lens epithelial cells to stress-induced apoptosis mainly through suppression of αB-crystallin expression.

### CREB directly regulates αB-crystallin gene

**Figure 7:**
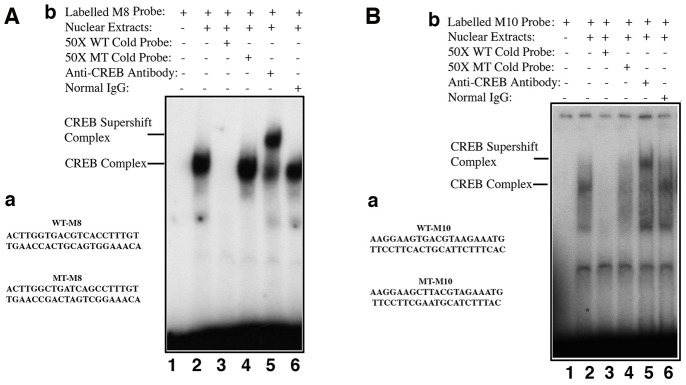
**Electrophoretic mobility shifting assays (EMSA) demonstrated that CREB directly binds to the promoter enhancer sequences of the αB-crystallin gene to control its expression.** Bioinformatics analysis revealed that the mouse αB-crystallin gene contains many half CREB binding sites in the proximal promoter, and completely conserved CREB sites in the upstream enhancer or downstream enhancer regions (Supplementary Figure 3). EMSA revealed that CREB can directly bind to the conserved CREB binding sites in both upstream (M8) or downstream (M10). **A**-a, diagram of the two oligos containing a well-conserved CREB binding site (WT-M8, top) or mutant CREB binding site (MT-M8, bottom), which were used for gel mobility shifting assays described in **A**-b. **A**-b, gel mobility shifting assays. Nuclear extracts prepared from pCI-CREB-αTN4-1 cells were incubated with γ-32P-ATP-labeled oligo-nucleotide containing wild-type CREB binding site (**A**-a, top) under various conditions shown in the figure. **B**-a, diagram of the two oligos containing a less conserved CREB binding site (WT-M10, top) or mutant CREB binding site (MT-M10, bottom), which were used for gel mobility shifting assays described in **B**-b. **B**-b, gel mobility shifting assays. Nuclear extracts prepared from pCI-CREB-αTN4-1 cells were incubated with γ-^32^P-ATP-labeled oligo-nucleotide containing wild-type M10 CREB binding site (**B**-a, top) under various conditions shown in the figure.

Next, we determined if CREB can directly regulate αB-crystallin gene. First, we used bioinformatics to search the CREB binding sites in αB-crystallin gene promoter. As shown in Supplementary Figure 3, the mouse αB-crystallin gene contains multiple copies of either well-conserved full CREB binding site such as M8 or the variated CREB full binding sites like M10 within the 250 kb sequences examined. Next, we tested if CREB can bind to these putative sites. We chose M8, the well conserved full CREB binding site as well as M10, the less conserved variant CREB binding sites (it has one nucleotide variation) as oligo probes to conduct gel mobility shifting assay. As shown in Figure 7A, 7B, nuclear extracts from pCI-CREB-αTN4-1 cells displayed strong binding to the M8 sequences, which can only be competed off by wild type but not mutant oligos. A much-reduced binding was observed when probe was derived from M10 site region. The authenticity of the CREB binding was confirmed by the formation of the supershifting bands after incubation with anti-CREB antibody (Figure 7A, 7B). Interestingly, we did not observe the supershifting band formation with the M1 oligos (Supplementary Figure 4B). Lack of the supershifting band may be due to the formation of heterodimers (see discussion). Together, our results suggest that different CREB binding sites in the αB-crystallin gene promoter and enhancer regions display differential affinities with CREB in the in vitro binding assays.

### CREB regulates αB-crystallin gene in vivo.

**Figure 8:**
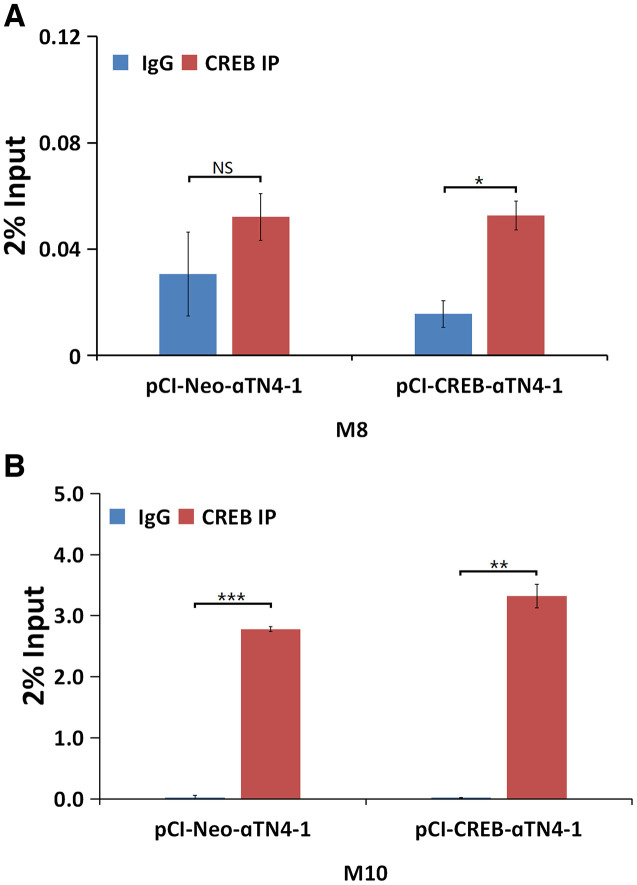
**ChIP assays to demonstrate that CREB binds to the promoter of αB-crystallin gene in vivo.** qChIP experiments revealed that in the in vivo condition, CREB displayed stronger affinity with M10 site (**B**), suggesting that CREB may interact with its partner to bind to the M10 site, and to a less degree, to the M8 site (**A**). All experiments were repeated three times. Error bar represents standard deviation, N=3. * p<0.05; ** p<0.01; *** p<0.005; NS, statistically not significant.

We next determined if CREB can regulate αB-crystallin gene in vivo. To do so, we conducted ChIP assays using the oligos (Supplementary Table 1) from the M8 and M10 regions. The nuclear extracts isolated from vector- or CREB-transfected cells were immuno-precipitated with mock IgG or anti-CREB antibody. The precipitated complexes were used for extraction of template DNAs, which were then amplified in QPCR analysis. As shown in Figure 8, both M8 and M10 cis-elements can be bound by CREB. Moreover, the M10 region seems to be bound by CREB more tightly in the exo vivo condition. ChIP assay also confirmed that CREB can bind to M1 region (Supplementary Figure 4C). These results demonstrated that CREB can bind to multiple sites of the enhancer regions of the αB-crystallin gene in vivo to suppress expression of the later.

## DISCUSSION

In the present study, we have obtained the following results: 1) In cultured rat lenses, oxidative stress-induced apoptosis appeared to be derived from downregulation of αB-crystallin expression which was associated with CREB upregulation; 2) RNAseq analysis, QRT-PCR and western blot analysis demonstrated that CREB-expressing cells displayed strongest sensitivity to stress-induced apoptosis, which is largely due to suppression of αB-crystallin expression; 3) Knockdown of CREB activates expression of the endogenous αB-crystallin and enhances its resistance to oxidative stress-induced apoptosis; 4) Over-expression of the exogenous human αB-crystallin cDNA can rescue the CREB-expressing cells from oxidative stress-induced apoptosis; 5) Both gel mobility shifting assay and ChIP analysis reveal that CREB directly suppresses expression of αB-crystallin gene in vivo. Thus, our results showed that CREB plays an important role in stress response. It sensitizes lens epithelial cells to stress-induced apoptosis by suppressing expression of the αB-crystallin gene.

### CREB negatively regulates cell survival of lens epithelial cells

It has been well established that CREB can promote survival. For example, in the nervous system, CREB has been shown to promote neuronal survival by NGF and BDGF [13, 25–27]. Through activation of RSK90 kinase, the activated CREB can promote Bcl-2 expression and thus enhance survival of the related neurons. In a more recent study, it was reported that the mammalian embryo-derived preimplantation factor (PIF) enables neuroprotection in rodent models of experimental autoimmune encephalomyelitis and perinatal brain injury [82]. Mechanistically, it was found that PIF can activate both PKA and PKC to phosphorylate CREB at S133, and the activated CREB can facilitate expressions of GAP43, BDNF besides Bcl-2 to exert the neuroprotection [82]. In the present study, we observed that CREB plays an important role in stress response. We demonstrated that in cultured rat lenses, oxidative stress-induced apoptosis was associated with downregulated expression of αB-crystallin, and the later was closely associated with CREB upregulation. Using CREB-transfected cell line, we further demonstrated that mouse lens epithelial cells expressing exogenous CREB were very sensitive to oxidative stress-induced apoptosis (Figure 3). More importantly, we found that overexpressed CREB in pCI-CREB-αTN4-1 cells significantly downregulates expression of the αB-crystallin gene (Figure 4). In contrast, silence of CREB stimulates expression of the αB-crystallin gene, which confers the resistance against oxidative stress-induced apoptosis (Figure 5). Furthermore, overexpression of the exogenous human αB-crystallin can largely rescue the pCI-CREB-αTN4-1 cells from oxidative stress-induced apoptosis (Figure 6). Why downregulation of αB-crystallin expression by CREB sensitizes the mouse lens epithelial cells to oxidative stress-induced apoptosis? It has been well established from numerous laboratories including ours that αB-crystallin is a strong anti-apoptotic regulator [53–75]. It represses apoptosis through several mechanisms. First, it can bind to both procaspase-3 and the intermediate of the partially processed caspase-3 to prevent procaspase-3 activation [62–64, 68]. Second, it can interact with Bax and Bcl-X_S_ to prevent translocation of the later into mitochondria, and thus shut off the activation of the intrinsic death pathway [68]. Third, it can interact with GRF2 to suppress the RAS-RAF-MEK-ERK signaling pathway which mediates both calcimycin and UVA-induced apoptosis [69–70]. Together, by suppressing αB-crystallin expression, CREB negatively regulates the viability of lens epithelial cells.

### CREB transcriptionally regulates expression of the αB-crystallin gene

Although CREB has been shown to positively regulate αA-crystallin [83–84], our results have demonstrated that CREB negatively regulates expression of αB-crystallin gene (Figures 4, 5, 7, 8). Bioinformatics analysis revealed that αB-crystallin gene contains both fully conserved CREB binding site such as M8, and also less conserved CREB binding sites like M1 and M10 (M1 has a changed nucleotide from G to C at the position 5, and M10 has a varied nucleotide from C to A at position 7) (Supplementary Figure 3). EMSA revealed that the CREB strongly binds to fully conserved M8 binding site, but displayed significant decrease in binding to the M10 site with one nucleotide variation in the 7^th^ position in the in vitro binding assays. Nevertheless, anti-CREB antibody can bind to the CREB-M8 complex or CREB-M10 complex to form the supershifting bands (Figure 7A and 7B). In contrast, the M1 site has a variated nucleotide in the middle, and this greatly affected CREB binding since anti-CREB antibody could not bind to the CREB-M1 complex to form the supershifting band (Supplementary Figure 4). Lack of the supershifting band may be due to the masking of the epitope for anti-CREB binding. We could not, however, rule out the possibility that the proteins bound to M1 site may be a heterodimer of CREB and AP-1 component, or other interacting transcription factor partner. CREB has been shown to interact with numerous other factors [85–86]. Our EMSA data showed that the genomic gene for αB-crystallin spans over 240 kb since both the M8 (-91 kb) and the M10 site (+150 kb) are functional CREB binding sites. The ChIP assay results with oligos derived from the M8 and M10 regions (Figure 8) also support out conclusion.

In summary, our results demonstrate that CREB is an important transcription factor mediating stress response, and it promotes stress-induced apoptosis by suppressing αB-crystallin expression.

## MATERIALS AND METHODS

### Chemicals

Various molecular biology reagents were purchased from Invitrogen Life Technologies, Gaithersburg, MD; Stratagene, La Jolla, CA and Promega Biotech, Madison, WI. All the oligos, DNA and protein size markers were purchased from Invitrogen Life Technologies, Gaithersburg, MD and Sangon Biotech (Shanghai) Co., Ltd. Various antibodies were obtained from Cell Signaling Technology, Boston, MA; abCam Inc., Cambridge, MA; Santa Cruz Biotechnology, Inc. Dallas, TX; Sigma-Aldrich, St. Louis, MO; Transduction Laboratories, San Diego, CA. The culture medium, and most other chemicals and antibiotics were purchased from Sigma-Aldrich, St. Louis, MO and Invitrogen Life Technologies, Gaithersburg, MD.

### Culture of mouse lens epithelial cells (αTN4-1)

The mouse lens epithelial cell line, αTN4-1**,** was kindly provided by Dr. Paul Russel of the National Eye Institute, and grown in Dulbecco’s Modified Eagle’s Medium (D7777, Sigma) containing 10% fetal bovine serum as described previously [87–88]. The medium was prepared in ion-exchanged double-distilled water to give an osmolarity of 300 ± 5 mosmols supplemented with 26 mM NaHCO_3_ and 50 units/ml penicillin and streptomycin. Media were sterilized by filtration through 0.22-μm filters with pH adjusted to 7.2. All cells were kept at 37 °C and 5% CO_2_ gas phase.

### Measurement of hydrogen peroxide and free thiol levels

The free thiol content was determined with a fluorometric thiol quantitation kit (Sigma-Aldrich Corp., #MAK151) according to the manufactory’s instruction. Briefly, rat lenses or αTN4-1 cells were treated with 40 mU GO for 0 to 6 hours. After GO treatment, the cells were washed with PBS for three times, lysed in 150 μl of the assay buffer, and 20 μl of the cell lysates were used for each assay reaction. For the rat lens, the epithelial cells from six rat lenses were lysed in 180 μl assay buffer, and 50 μl lysates were used for each assay reaction.

### Preparation of expression constructs

The CREB cDNA was cloned into pCI-Neo vector at EcoRI and XbaI sites. Human αB cDNA was amplified by RT-PCR from human lens mRNA using the following primers: 5’-TACCTCGAGATGGACATCGCCATCCAC-3’ (forward), 5’-CAACCCGGGTTCAAGAAAGGGCATCTA-3’ (reverse) as described before [68]. The cDNA was further inserted into an enhanced green fluorescence protein expression vector, pEGFPC3, at the XhoI and SmaI sites that were created by PCR to generate in frame fusion constructs. To target CREB knockout, the CRISPR/Cas9 construct was prepared with the oligos 5’-caccgtttcaaggaggccttcctac-3’ and 5’-aaacgtaggaaggcctccttgaaac-3’ annealed and inserted into pSpCas9(BB)-2A-Puro (PX459) vector. The knockout results were verified by DNA sequencing and western blot analysis (Figure 5A –5C)

### Establishment of stable expression cell lines

The pCI-Neo and pCI-CREB constructs were amplified in DH-5α and purified by two rounds of CsCl ultracentrifugation as previously described [68, 70]. Transfection of αTN4-1 cells was performed using Lipofectamine^TM^ 2000 from the Invitrogen Life Technologies according to the company instruction manual. The pCI-Neo and pCI-CREB transfected cells were then subjected to G418 (500 μg/ml) selection for 4-6 weeks and subsequently individual clones for the following stable transfected cell lines were established. These include pCI-αTN4-1 and pCI-CREB-αTN4-1.

### Treatment by glucose oxidase

The αTN4-1 cells were grown to 90% confluence in DMEM containing 10% fetal bovine serum [68, 70]. Then, the media with serum-free plus 40mU glucose oxidase (GO) were used to replace the culture media for the required period of incubation as indicated. After treatment, all samples were collected for analysis of apoptosis and gene expression.

### Apoptosis analysis with cellTiter-Glo^®^ luminescent cell viability assay and live/dead viability/cytotoxicity

The percentage of apoptotic cells was determined either by cellTiter-Glo^®^ luminescent cell viability assay kit (G7573, Promega) [89] or using live/dead viability/cytotoxicity kit (L3224, Thermofish Scientific) according to the company instruction. The CellTiter-Glo® Luminescent Cell Viability Assay is a homogeneous method to determine the number of viable cells in culture based on quantitation of the ATP present, which signals the presence of metabolically active cells. About 2x10^4^ cells were seeded into each well of 96-well plates, 12h later, the culture media were replaced with 100ul medium containing 40 mU GO to induce cell apoptosis. After treatment, the same volumes of the mixed CellTiter-Glo® Buffer and CellTiter-Glo® Substrate were added into each well and luminescence was read by synergy microplate reader (BioTek).

### RT-PCR, qPCR and RNAseq

RT-PCR and qPCR were conducted as we described previously [87–88]. Total RNAs were extracted using the TRIzol Reagent (Invitrogen). cDNA synthesis was performed with 1 μg of total RNAs using the HiScript II Q RT SuperMix for qPCR (+gDNA wiper) kit (R223-01; Vazyme). Gene expression levels were analyzed using ChamQ SYBR Color qPCR Master Mix (Q411-02; Vazyme) and the LightCycler 480 qPCR system (Roche). The assays were performed in triplicate, and the Ct values were normalized to β-actin. The primers used are listed in Supplementary Table 1.

For the RNAseq analyses, total RNAs were extracted from pCI-αTN4-1 and pCI-CREB-αTN4-1 cells using the TRIzol reagent according to the manufacturer’s instruction. Preparation of the RNAseq library and subsequent sequencing were conducted by the Berry Genomics Corporation. Pooled samples of two biological repeats were sequenced on Illumina Nova 6000. The obtained sequence reads were cleaned and mapped to (GRCm38/mm10) using Tophat. Gene expression and changes were analyzed using Bowtie2 and RSEM. The relative abundance of mRNAs was normalized and presented as fragments per kilobase of transcript per million mapped reads (FPKM). Hierarchical cluster and scatter plot analyses of gene expression levels were performed using the R software (http://www.r-project.org/). KEGG analysis was carried out by Kobas. Samples harvested from two independent experiments were pooled and used for each RNAseq analysis sample.

### Protein preparation and western blotting analysis

A total of 80 rats of 4-week including both male and female supplied by the Sun Yat-sen University Animal Facility were used for GO treatment study. Animal usage was strictly conducted according to the animal usage protocol approved by the IACUC Committee of Sun Yat-sen University. Rat lenses were dissected as previously described [31, 32]. The dissected lenses were first incubated in medium 199 at 37^o^C overnight to exclude damaged (becoming opaque) lenses, and the selected transparent lenses were then treated with 40 mU glucose oxidase (GO) for 0 to 180 minutes in medium 199 at 37^o^C incubation. After GO treatment, both mock and GO-treated lenses were dissected into epithelial cells and fiber cells which were used for extraction of total proteins as described below.

For cultured cells, total proteins were prepared from the mock, or 40mU glucose oxidase-treated αTN4-1, pCI-αTN4-1, or pCI-CREB-αTN4-1 cells for 3 to 6 hours. After treatment, total proteins were extracted using protein extraction buffer in the presence of the protease inhibitor cocktail. The buffer contained 1% NP-40, 0.5% sodium deoxycholate, 0.1% SDS, 9.1 mM Na_2_HPO_4_, 1.7 mM NaH_2_PO_4_, 150 mM NaCl, 30 μl/ml aprotinin with pH of the preparation adjusted to 7.4. After homogenization by passing through an initial 18.5-gauge needle followed by the 23.5G needle, the cell lysate was centrifuged at 10,000 x *g* for 20 min at 4^o^C, the supernatant fraction of each sample was collected and stored in aliquots at -80^o^C. Fifty or one hundred micrograms of total proteins in each sample were resolved by 10 % SDS-polyacrylamide gel and transferred into supported nitrocellulose membranes. The protein blots were blocked with 5% nonfat milk in TBS (10 mM Tris HCl, pH8.0/ 150 mM NaCl) for one hour, then incubated overnight at 4^o^C with following primary antibodies: anti-CREB (4820), phospho-CREB at S133 (9198) antibodies from Cell Signaling Inc., anti-αB-crystallin antibody (generous gift of Dr. J Horwitz in the Julie Eye Institute of UCLA), and anti-β-actin, anti-GAPDH as well as anti-tubulin antibodies (Sigma) at a dilution of 1 to 500 to 2,000 (μg/ml) in 5% milk prepared in TBS (for total proteins) or 5% BSA in TBS (for phosphor-antibody). The secondary antibody is anti-mouse IgG or anti-rabbit IgG at a dilution of 1 to 1,000 (Amersham). Immunoreactivity was detected with an enhanced chemilluminescence detection kit according to the company's instruction (ECL, Amersham Corp.).

### Gel mobility shifting assays

The gel mobility shifting assay (EMSA) was conducted as we described before [90–92]. The oligos used were listed in Figure 7A, 7B and Supplementary Figure 4. For the binding assays, 2 μg of nuclear extracts from pCI-αTN4-1 or pCI-CREB-αTN4-1 cells were incubated with 1 x 10^5^ cpm of ^32^P-labeled double-stranded synthetic oligos for 20 min on ice. For competition experiments, 50-fold of the unlabeled wild type (WT) or mutant (MT) oligos were pre-incubated with the nuclear extracts for 20 min before addition of the labeled probe. For supershifting assays, the nuclear extracts were incubated with anti-CREB or normal IgG for 20 minutes on ice, then the reaction mixture was incubated with the labelled oligos for 20 min at room temperature, allowing formation of the supershifting complex. The reaction mixture was separated with 3.5% native gel.

### ChIP assays

The binding of CREB to the αB-crystallin gene distal enhancer sites was confirmed using SimpleChIP® Enzymatic Chromatin IP Kit (Magnetic Beads) (#9003, Cell Signaling), according to the manufacturer’s instruction. In brief, cells were grown to 95% confluence, approximately 3.0 x 10^7^ cells were incubated with 1% formaldehyde for 10 min at room temperature for crosslinking, which was terminated by addition of glycine solution. The cells were further washed with cold PBS twice and then scraped into cold PBS containing protease inhibitor cocktail. The pelleted cells were used for nuclei preparation and chromatin digestion. The nuclei lysates were sonicated 15 times for 10 s each time to generate DNA fragments that ranged in size from 200 to 1,000 bp. The sheared chromatin-lysates were incubated with either 5 μg of anti-histone 3, 5 μg of anti-CREB antibody or 5 μg of normal IgG overnight at 4°C, and then incubated for an additional 2 h with 30 μL protein G magnetic beads. The immunoprecipitates were washed by low salt wash buffer three times and high salt wash buffer one time, then suspended in the elution buffer, reverse cross-links by adding 6 μl 5M NaCl and 2 μl Proteinase K, and incubate 2 h at 65°C. Finally, these samples were processed for DNA purification using spin columns. The extracted DNA with specific primers listed in the Supplementary Table 1 were used for ChIP-qPCR assays.

### Immunofluorescence

Cells were seeded on Millicell EZ 24-well glass slides (Millipore). After PBS wash, cells were fixed with 4% paraformaldehyde, permeabilized with methanol/acetone 1:1, and blocked with normal rabbit or goat serum. Then the slides were incubated with the anti-CREB (#MA1-083, Invitrogen Inc.) and anti-p-CREB antibody (#9198, Cell Signaling Technology) or normal rabbit IgG at 4°C overnight. After the PBS washings, the slides were incubated with fluorescence goat anti-rabbit IgG or fluorescence goat anti-mouse IgG (1:200, Cell Signaling Technology). Cell nuclei were stained with 50 ng/ml 4’,6-diamidino-2-phenylindole (DAPI) for 5 min. Slides were mounted with anti-fade fluorescent mounting medium (Applygen). Images were acquired by a Zeiss 800 confocal microscope (CLSM, Carl Zeiss, Germany) and processed by ZEN software.

### Statistical analysis

All experiments were repeated at least three times (N=3) except for RNAseq analysis in which each analyzed sample was a pool of two separated samples (N=4). Significance was determined by two-tailed Student’s t-test [87, 88]. The error bar in all figures represents standard deviation.

## Supplementary Material

Supplementary Figures

Supplementary Table 1

## References

[1] Chandrasekaran A, Idelchik MD, Melendez JA. Redox control of senescence and age-related disease. Redox Biol. 2017; 11:91–102. 10.1016/j.redox.2016.11.005 27889642 PMC5126126

[2] Giorgio M, Trinei M, Migliaccio E, Pelicci PG. Hydrogen peroxide: a metabolic by-product or a common mediator of ageing signals? Nat Rev Mol Cell Biol. 2007; 8:722–28. 10.1038/nrm2240 17700625

[3] Spector A, Garner WH. Hydrogen peroxide and human cataract. Exp Eye Res. 1981; 33:673–81. 10.1016/s0014-4835(81)80107-8 7318962

[4] Spector A. Oxidative stress-induced cataract: mechanism of action. FASEB J. 1995; 9:1173–82. 7672510

[5] Mayr B, Montminy M. Transcriptional regulation by the phosphorylation-dependent factor CREB. Nat Rev Mol Cell Biol. 2001; 2:599–609. 10.1038/35085068 11483993

[6] Montminy MR, Sevarino KA, Wagner JA, Mandel G, Goodman RH. Identification of a cyclic-AMP-responsive element within the rat somatostatin gene. Proc Natl Acad Sci USA. 1986; 83:6682–86. 10.1073/pnas.83.18.6682 2875459 PMC386573

[7] Comb M, Birnberg NC, Seasholtz A, Herbert E, Goodman HM. A cyclic AMP- and phorbol ester-inducible DNA element. Nature. 1986; 323:353–56. 10.1038/323353a0 3020428

[8] Chrivia JC, Kwok RP, Lamb N, Hagiwara M, Montminy MR, Goodman RH. Phosphorylated CREB binds specifically to the nuclear protein CBP. Nature. 1993; 365:855–59. 10.1038/365855a0 8413673

[9] Arany Z, Sellers WR, Livingston DM, Eckner R. E1A-associated p300 and CREB-associated CBP belong to a conserved family of coactivators. Cell. 1994; 77:799–800. 10.1016/0092-8674(94)90127-9 8004670

[10] Ebrahimi A, Sevinç K, Gürhan Sevinç G, Cribbs AP, Philpott M, Uyulur F, Morova T, Dunford JE, Göklemez S, Ari Ş, Oppermann U, Önder TT. Bromodomain inhibition of the coactivators CBP/EP300 facilitate cellular reprogramming. Nat Chem Biol. 2019; 15:519–28. 10.1038/s41589-019-0264-z 30962627 PMC6504645

[11] Del Blanco B, Guiretti D, Tomasoni R, Lopez-Cascales MT, Muñoz-Viana R, Lipinski M, Scandaglia M, Coca Y, Olivares R, Valor LM, Herrera E, Barco A. CBP and SRF co-regulate dendritic growth and synaptic maturation. Cell Death Differ. 2019; 26:2208–22. 10.1038/s41418-019-0285-x 30850733 PMC6889142

[12] Yamanaka R, Shindo Y, Hotta K, Suzuki K, Oka K. GABA-induced intracellular mg^2+^ mobilization integrates and coordinates cellular information processing for the maturation of neural networks. Curr Biol. 2018; 28:3984–91.e5. 10.1016/j.cub.2018.10.044 30528584

[13] Cohen SM, Suutari B, He X, Wang Y, Sanchez S, Tirko NN, Mandelberg NJ, Mullins C, Zhou G, Wang S, Kats I, Salah A, Tsien RW, Ma H. Calmodulin shuttling mediates cytonuclear signaling to trigger experience-dependent transcription and memory. Nat Commun. 2018; 9:2451. 10.1038/s41467-018-04705-8 29934532 PMC6015085

[14] Hagiwara M, Brindle P, Harootunian A, Armstrong R, Rivier J, Vale W, Tsien R, Montminy MR. Coupling of hormonal stimulation and transcription via the cyclic AMP-responsive factor CREB is rate limited by nuclear entry of protein kinase a. Mol Cell Biol. 1993; 13:4852–59. 10.1128/mcb.13.8.4852 8336722 PMC360117

[15] Bartsch D, Casadio A, Karl KA, Serodio P, Kandel ER. CREB1 encodes a nuclear activator, a repressor, and a cytoplasmic modulator that form a regulatory unit critical for long-term facilitation. Cell. 1998; 95:211–23. 10.1016/s0092-8674(00)81752-3 9790528

[16] Altarejos JY, Montminy M. CREB and the CRTC co-activators: sensors for hormonal and metabolic signals. Nat Rev Mol Cell Biol. 2011; 12:141–51. 10.1038/nrm3072 21346730 PMC4324555

[17] Kim HJ, Hur SW, Park JB, Seo J, Shin JJ, Kim SY, Kim MH, Han DH, Park JW, Park JM, Kim SJ, Chun YS. Histone demethylase PHF2 activates CREB and promotes memory consolidation. EMBO Rep. 2019; 20:e45907. 10.15252/embr.201845907 31359606 PMC6726911

[18] Joy MT, Ben Assayag E, Shabashov-Stone D, Liraz-Zaltsman S, Mazzitelli J, Arenas M, Abduljawad N, Kliper E, Korczyn AD, Thareja NS, Kesner EL, Zhou M, Huang S, et al. CCR5 is a therapeutic target for recovery after stroke and traumatic brain injury. Cell. 2019; 176:1143–57.e13. 10.1016/j.cell.2019.01.044 30794775 PMC7259116

[19] Bourtchuladze R, Frenguelli B, Blendy J, Cioffi D, Schutz G, Silva AJ. Deficient long-term memory in mice with a targeted mutation of the cAMP-responsive element-binding protein. Cell. 1994; 79:59–68. 10.1016/0092-8674(94)90400-6 7923378

[20] Yin JC, Wallach JS, Del Vecchio M, Wilder EL, Zhou H, Quinn WG, Tully T. Induction of a dominant negative CREB transgene specifically blocks long-term memory in drosophila. Cell. 1994; 79:49–58. 10.1016/0092-8674(94)90399-9 7923376

[21] Yin JC, Del Vecchio M, Zhou H, Tully T. CREB as a memory modulator: induced expression of a dCREB2 activator isoform enhances long-term memory in drosophila. Cell. 1995; 81:107–15. 10.1016/0092-8674(95)90375-5 7720066

[22] Caracciolo L, Marosi M, Mazzitelli J, Latifi S, Sano Y, Galvan L, Kawaguchi R, Holley S, Levine MS, Coppola G, Portera-Cailliau C, Silva AJ, Carmichael ST. CREB controls cortical circuit plasticity and functional recovery after stroke. Nat Commun. 2018; 9:2250. 10.1038/s41467-018-04445-9 29884780 PMC5993731

[23] Maeder CI, Kim JI, Liang X, Kaganovsky K, Shen A, Li Q, Li Z, Wang S, Xu XZ, Li JB, Xiang YK, Ding JB, Shen K. The THO complex coordinates transcripts for synapse development and dopamine neuron survival. Cell. 2018; 174:1436–49.e20. 10.1016/j.cell.2018.07.046 30146163 PMC6542560

[24] Rao-Ruiz P, Couey JJ, Marcelo IM, Bouwkamp CG, Slump DE, Matos MR, van der Loo RJ, Martins GJ, van den Hout M, van IJcken WF, Costa RM, van den Oever MC, Kushner SA. Engram-specific transcriptome profiling of contextual memory consolidation. Nat Commun. 2019; 10:2232. 10.1038/s41467-019-09960-x 31110186 PMC6527697

[25] Riccio A, Ahn S, Davenport CM, Blendy JA, Ginty DD. Mediation by a CREB family transcription factor of NGF-dependent survival of sympathetic neurons. Science. 1999; 286:2358–61. 10.1126/science.286.5448.2358 10600750

[26] Bonni A, Brunet A, West AE, Datta SR, Takasu MA, Greenberg ME. Cell survival promoted by the ras-MAPK signaling pathway by transcription-dependent and -independent mechanisms. Science. 1999; 286:1358–62. 10.1126/science.286.5443.1358 10558990

[27] Merk DJ, Ohli J, Merk ND, Thatikonda V, Morrissy S, Schoof M, Schmid SN, Harrison L, Filser S, Ahlfeld J, Erkek S, Raithatha K, Andreska T, et al. Opposing effects of CREBBP mutations govern the phenotype of rubinstein-taybi syndrome and adult SHH medulloblastoma. Dev Cell. 2018; 44:709–24.e6. 10.1016/j.devcel.2018.02.012 29551561

[28] Xing J, Ginty DD, Greenberg ME. Coupling of the RAS-MAPK pathway to gene activation by RSK2, a growth factor-regulated CREB kinase. Science. 1996; 273:959–63. 10.1126/science.273.5277.959 8688081

[29] Rudolph D, Tafuri A, Gass P, Hämmerling GJ, Arnold B, Schütz G. Impaired fetal T cell development and perinatal lethality in mice lacking the cAMP response element binding protein. Proc Natl Acad Sci USA. 1998; 95:4481–86. 10.1073/pnas.95.8.4481 9539763 PMC22515

[30] Long F, Schipani E, Asahara H, Kronenberg H, Montminy M. The CREB family of activators is required for endochondral bone development. Development. 2001; 128:541–50. 11171337 10.1242/dev.128.4.541

[31] Li WC, Kuszak JR, Dunn K, Wang RR, Ma W, Wang GM, Spector A, Leib M, Cotliar AM, Weiss M. Lens epithelial cell apoptosis appears to be a common cellular basis for non-congenital cataract development in humans and animals. J Cell Biol. 1995; 130:169–81. 10.1083/jcb.130.1.169 7790371 PMC2120521

[32] Li WC, Spector A. Lens epithelial cell apoptosis is an early event in the development of UVB-induced cataract. Free Radic Biol Med. 1996; 20:301–11. 10.1016/0891-5849(96)02050-3 8720900

[33] Sax CM, Piatigorsky J. Expression of the alpha-Crystallin/small heat-shock protein/molecular chaperone genes in the lens and other tissues. Adv Enzymol Relat Areas Mol Biol. 1994; 69:155–201. 10.1002/9780470123157.ch5 7817868

[34] Horwitz J. The function of alpha-crystallin in vision. Semin Cell Dev Biol. 2000; 11:53–60. 10.1006/scdb.1999.0351 10736264

[35] Ingolia TD, Craig EA. Four small drosophila heat shock proteins are related to each other and to mammalian alpha-crystallin. Proc Natl Acad Sci USA. 1982; 79:2360–64. 10.1073/pnas.79.7.2360 6285380 PMC346193

[36] Horwitz J. Alpha-crystallin can function as a molecular chaperone. Proc Natl Acad Sci USA. 1992; 89:10449–53. 10.1073/pnas.89.21.10449 1438232 PMC50356

[37] Bhat SP, Nagineni CN. Alpha B subunit of lens-specific protein alpha-crystallin is present in other ocular and non-ocular tissues. Biochem Biophys Res Commun. 1989; 158:319–25. 10.1016/s0006-291x(89)80215-3 2912453

[38] Iwaki T, Kume-Iwaki A, Liem RK, Goldman JE. Alpha b-crystallin is expressed in non-lenticular tissues and accumulates in alexander’s disease brain. Cell. 1989; 57:71–78. 10.1016/0092-8674(89)90173-6 2539261

[39] Dubin RA, Wawrousek EF, Piatigorsky J. Expression of the murine alpha b-crystallin gene is not restricted to the lens. Mol Cell Biol. 1989; 9:1083–91. 10.1128/mcb.9.3.1083 2725488 PMC362698

[40] Rao PV, Horwitz J, Zigler JS Jr. Alpha-crystallin, a molecular chaperone, forms a stable complex with carbonic anhydrase upon heat denaturation. Biochem Biophys Res Commun. 1993; 190:786–93. 10.1006/bbrc.1993.1118 8094957

[41] Kelley MJ, David LL, Iwasaki N, Wright J, Shearer TR. Alpha-crystallin chaperone activity is reduced by calpain II in vitro and in selenite cataract. J Biol Chem. 1993; 268:18844–49. 8395520

[42] Boyle D, Takemoto L. Characterization of the alpha-gamma and alpha-beta complex: evidence for an in vivo functional role of alpha-crystallin as a molecular chaperone. Exp Eye Res. 1994; 58:9–15. 10.1006/exer.1994.1190 8157104

[43] Nicholl ID, Quinlan RA. Chaperone activity of alpha-crystallins modulates intermediate filament assembly. EMBO J. 1994; 13:945–53. 7906647 10.1002/j.1460-2075.1994.tb06339.x PMC394896

[44] Clark JI, Huang QL. Modulation of the chaperone-like activity of bovine alpha-crystallin. Proc Natl Acad Sci USA. 1996; 93:15185–89. 10.1073/pnas.93.26.15185 8986785 PMC26378

[45] Sun TX, Das BK, Liang JJ. Conformational and functional differences between recombinant human lens alphaA- and alphaB-crystallin. J Biol Chem. 1997; 272:6220–25. 10.1074/jbc.272.10.6220 9045637

[46] Reddy GB, Das KP, Petrash JM, Surewicz WK. Temperature-dependent chaperone activity and structural properties of human alphaA- and alphaB-crystallins. J Biol Chem. 2000; 275:4565–70. 10.1074/jbc.275.7.4565 10671481

[47] Cobb BA, Petrash JM. Structural and functional changes in the alpha a-crystallin R116C mutant in hereditary cataracts. Biochemistry. 2000; 39:15791–98. 10.1021/bi001453j 11123904 PMC2902970

[48] Bova MP, Yaron O, Huang Q, Ding L, Haley DA, Stewart PL, Horwitz J. Mutation R120G in alphaB-crystallin, which is linked to a desmin-related myopathy, results in an irregular structure and defective chaperone-like function. Proc Natl Acad Sci USA. 1999; 96:6137–42. 10.1073/pnas.96.11.6137 10339554 PMC26848

[49] Derham BK, van Boekel MA, Muchowski PJ, Clark JI, Horwitz J, Hepburne-Scott HW, de Jong WW, Crabbe MJ, Harding JJ. Chaperone function of mutant versions of alpha a- and alpha b-crystallin prepared to pinpoint chaperone binding sites. Eur J Biochem. 2001; 268:713–21. 10.1046/j.1432-1327.2001.01929.x 11168410

[50] Takemoto L. Release of alpha-a sequence 158-173 correlates with a decrease in the molecular chaperone properties of native alpha-crystallin. Exp Eye Res. 1994; 59:239–42. 10.1006/exer.1994.1103 7835414

[51] Shroff NP, Cherian-Shaw M, Bera S, Abraham EC. Mutation of R116C results in highly oligomerized alpha a-crystallin with modified structure and defective chaperone-like function. Biochemistry. 2000; 39:1420–26. 10.1021/bi991656b 10684623

[52] Kantorow M, Piatigorsky J. Alpha-crystallin/small heat shock protein has autokinase activity. Proc Natl Acad Sci USA. 1994; 91:3112–16. 10.1073/pnas.91.8.3112 8159713 PMC43525

[53] Mehlen P, Preville X, Chareyron P, Briolay J, Klemenz R, Arrigo AP. Constitutive expression of human hsp27, drosophila hsp27, or human alpha b-crystallin confers resistance to TNF- and oxidative stress-induced cytotoxicity in stably transfected murine L929 fibroblasts. J Immunol. 1995; 154:363–74. 7995955

[54] Mehlen P, Schulze-Osthoff K, Arrigo AP. Small stress proteins as novel regulators of apoptosis. Heat shock protein 27 blocks fas/APO-1- and staurosporine-induced cell death. J Biol Chem. 1996; 271:16510–14. 10.1074/jbc.271.28.16510 8663291

[55] Mehlen P, Kretz-Remy C, Préville X, Arrigo AP. Human hsp27, drosophila hsp27 and human alphaB-crystallin expression-mediated increase in glutathione is essential for the protective activity of these proteins against TNFalpha-induced cell death. EMBO J. 1996; 15:2695–706. 8654367 PMC450205

[56] Martin JL, Mestril R, Hilal-Dandan R, Brunton LL, Dillmann WH. Small heat shock proteins and protection against ischemic injury in cardiac myocytes. Circulation. 1997; 96:4343–48. 10.1161/01.cir.96.12.4343 9416902

[57] Andley UP, Song Z, Wawrousek EF, Bassnett S. The molecular chaperone alphaA-crystallin enhances lens epithelial cell growth and resistance to UVA stress. J Biol Chem. 1998; 273:31252–61. 10.1074/jbc.273.47.31252 9813033

[58] Andley UP, Song Z, Wawrousek EF, Fleming TP, Bassnett S. Differential protective activity of alpha a- and alphaB-crystallin in lens epithelial cells. J Biol Chem. 2000; 275:36823–31. 10.1074/jbc.M004233200 10967101

[59] Hoover HE, Thuerauf DJ, Martindale JJ, Glembotski CC. Alpha b-crystallin gene induction and phosphorylation by MKK6-activated p38. A potential role for alpha b-crystallin as a target of the p38 branch of the cardiac stress response. J Biol Chem. 2000; 275:23825–33. 10.1074/jbc.M003864200 10816593

[60] Ray PS, Martin JL, Swanson EA, Otani H, Dillmann WH, Das DK. Transgene overexpression of alphaB crystallin confers simultaneous protection against cardiomyocyte apoptosis and necrosis during myocardial ischemia and reperfusion. FASEB J. 2001; 15:393–402. 10.1096/fj.00-0199com 11156955

[61] Li DW, Xiang H, Mao YW, Wang J, Fass U, Zhang XY, Xu C. Caspase-3 is actively involved in okadaic acid-induced lens epithelial cell apoptosis. Exp Cell Res. 2001; 266:279–91. 10.1006/excr.2001.5223 11399056

[62] Mao YW, Xiang H, Wang J, Korsmeyer S, Reddan J, Li DW. Human bcl-2 gene attenuates the ability of rabbit lens epithelial cells against H2O2-induced apoptosis through down-regulation of the alpha b-crystallin gene. J Biol Chem. 2001; 276:43435–45. 10.1074/jbc.M102195200 11546795

[63] Kamradt MC, Chen F, Cryns VL. The small heat shock protein alpha b-crystallin negatively regulates cytochrome C- and caspase-8-dependent activation of caspase-3 by inhibiting its autoproteolytic maturation. J Biol Chem. 2001; 276:16059–63. 10.1074/jbc.C100107200 11274139

[64] Kamradt MC, Chen F, Sam S, Cryns VL. The small heat shock protein alpha b-crystallin negatively regulates apoptosis during myogenic differentiation by inhibiting caspase-3 activation. J Biol Chem. 2002; 277:38731–36. 10.1074/jbc.M201770200 12140279

[65] Andley UP, Patel HC, Xi JH. The R116C mutation in alpha a-crystallin diminishes its protective ability against stress-induced lens epithelial cell apoptosis. J Biol Chem. 2002; 277:10178–86. 10.1074/jbc.M109211200 11756414

[66] Alge CS, Priglinger SG, Neubauer AS, Kampik A, Zillig M, Bloemendal H, Welge-Lussen U. Retinal pigment epithelium is protected against apoptosis by alphaB-crystallin. Invest Ophthalmol Vis Sci. 2002; 43:3575–82. 12407170

[67] Morrison LE, Hoover HE, Thuerauf DJ, Glembotski CC. Mimicking phosphorylation of alphaB-crystallin on serine-59 is necessary and sufficient to provide maximal protection of cardiac myocytes from apoptosis. Circ Res. 2003; 92:203–11. 10.1161/01.res.0000052989.83995.a5 12574148

[68] Mao YW, Liu JP, Xiang H, Li DW. Human alphaA- and alphaB-crystallins bind to bax and bcl-X(S) to sequester their translocation during staurosporine-induced apoptosis. Cell Death Differ. 2004; 11:512–26. 10.1038/sj.cdd.4401384 14752512

[69] Liu JP, Schlosser R, Ma WY, Dong Z, Feng H, Lui L, Huang XQ, Liu Y, Li DW. Human alphaA- and alphaB-crystallins prevent UVA-induced apoptosis through regulation of PKCalpha, RAF/MEK/ERK and AKT signaling pathways. Exp Eye Res. 2004; 79:393–403. 15669141

[70] Li DW, Liu JP, Mao YW, Xiang H, Wang J, Ma WY, Dong Z, Pike HM, Brown RE, Reed JC. Calcium-activated RAF/MEK/ERK signaling pathway mediates p53-dependent apoptosis and is abrogated by alpha b-crystallin through inhibition of RAS activation. Mol Biol Cell. 2005; 16:4437–53. 10.1091/mbc.e05-01-0010 16000378 PMC1196350

[71] Maloyan A, Sanbe A, Osinska H, Westfall M, Robinson D, Imahashi K, Murphy E, Robbins J. Mitochondrial dysfunction and apoptosis underlie the pathogenic process in alpha-B-crystallin desmin-related cardiomyopathy. Circulation. 2005; 112:3451–61. 10.1161/CIRCULATIONAHA.105.572552 16316967 PMC1398051

[72] Kamradt MC, Lu M, Werner ME, Kwan T, Chen F, Strohecker A, Oshita S, Wilkinson JC, Yu C, Oliver PG, Duckett CS, Buchsbaum DJ, LoBuglio AF, et al. The small heat shock protein alpha b-crystallin is a novel inhibitor of TRAIL-induced apoptosis that suppresses the activation of caspase-3. J Biol Chem. 2005; 280:11059–66. 10.1074/jbc.M413382200 15653686

[73] Morozov V, Wawrousek EF. Caspase-dependent secondary lens fiber cell disintegration in alphaA-/alphaB-crystallin double-knockout mice. Development. 2006; 133:813–21. 10.1242/dev.02262 16439475

[74] Ousman SS, Tomooka BH, van Noort JM, Wawrousek EF, O’Connor KC, Hafler DA, Sobel RA, Robinson WH, Steinman L. Protective and therapeutic role for alphaB-crystallin in autoimmune demyelination. Nature. 2007; 448:474–79. 10.1038/nature05935 17568699

[75] Yan Q, Liu JP, Li DW. Apoptosis in lens development and pathology. Differentiation. 2006; 74:195–211. 10.1111/j.1432-0436.2006.00068.x 16759286

[76] Mercatelli N, Dimauro I, Ciafré SA, Farace MG, Caporossi D. AlphaB-crystallin is involved in oxidative stress protection determined by VEGF in skeletal myoblasts. Free Radic Biol Med. 2010; 49:374–82. 10.1016/j.freeradbiomed.2010.04.027 20441791

[77] Kannan R, Sreekumar PG, Hinton DR. Novel roles for α-crystallins in retinal function and disease. Prog Retin Eye Res. 2012; 31:576–604. 10.1016/j.preteyeres.2012.06.001 22721717 PMC3472046

[78] Dou G, Sreekumar PG, Spee C, He S, Ryan SJ, Kannan R, Hinton DR. Deficiency of αB crystallin augments ER stress-induced apoptosis by enhancing mitochondrial dysfunction. Free Radic Biol Med. 2012; 53:1111–22. 10.1016/j.freeradbiomed.2012.06.042 22781655 PMC3454510

[79] Christopher KL, Pedler MG, Shieh B, Ammar DA, Petrash JM, Mueller NH. Alpha-crystallin-mediated protection of lens cells against heat and oxidative stress-induced cell death. Biochim Biophys Acta. 2014; 1843:309–15. 10.1016/j.bbamcr.2013.11.010 24275510 PMC3901642

[80] Bódi B, Tóth EP, Nagy L, Tóth A, Mártha L, Kovács Á, Balla G, Kovács T, Papp Z. Titin isoforms are increasingly protected against oxidative modifications in developing rat cardiomyocytes. Free Radic Biol Med. 2017; 113:224–35. 10.1016/j.freeradbiomed.2017.09.015 28943453

[81] Dimauro I, Antonioni A, Mercatelli N, Grazioli E, Fantini C, Barone R, Macaluso F, Di Felice V, Caporossi D. The early response of αB-crystallin to a single bout of aerobic exercise in mouse skeletal muscles depends upon fiber oxidative features. Redox Biol. 2019; 24:101183. 10.1016/j.redox.2019.101183 30974319 PMC6454247

[82] Mueller M, Schoeberlein A, Zhou J, Joerger-Messerli M, Oppliger B, Reinhart U, Bordey A, Surbek D, Barnea ER, Huang Y, Paidas M. PreImplantation factor bolsters neuroprotection via modulating protein kinase a and protein kinase C signaling. Cell Death Differ. 2015; 22:2078–86. 10.1038/cdd.2015.55 25976303 PMC4816111

[83] Yang Y, Stopka T, Golestaneh N, Wang Y, Wu K, Li A, Chauhan BK, Gao CY, Cveklová K, Duncan MK, Pestell RG, Chepelinsky AB, Skoultchi AI, Cvekl A. Regulation of alphaA-crystallin via Pax6, c-maf, CREB and a broad domain of lens-specific chromatin. EMBO J. 2006; 25:2107–18. 10.1038/sj.emboj.7601114 16675956 PMC1462985

[84] Cvekl A, Kashanchi F, Sax CM, Brady JN, Piatigorsky J. Transcriptional regulation of the mouse alpha a-crystallin gene: activation dependent on a cyclic AMP-responsive element (DE1/CRE) and a pax-6-binding site. Mol Cell Biol. 1995; 15:653–60. 10.1128/mcb.15.2.653 7823934 PMC231924

[85] De Falco V, Tamburrino A, Ventre S, Castellone MD, Malek M, Manié SN, Santoro M. CD44 proteolysis increases CREB phosphorylation and sustains proliferation of thyroid cancer cells. Cancer Res. 2012; 72:1449–58. 10.1158/0008-5472.CAN-11-3320 22271686

[86] David-Watine B, Yaniv M. Two RAREs and an overlapping CRE are involved in the hepatic transcriptional regulation of the Q10 MHC class I gene. Cell Death Differ. 1996; 3:37–46. 17180053

[87] Gong L, Liu F, Xiong Z, Qi R, Luo Z, Gong X, Nie Q, Sun Q, Liu YF, Qing W, Wang L, Zhang L, Tang X, et al. Heterochromatin protects retinal pigment epithelium cells from oxidative damage by silencing p53 target genes. Proc Natl Acad Sci USA. 2018; 115:E3987–95. 10.1073/pnas.1715237115 29622681 PMC5924883

[88] Sun Q, Gong L, Qi R, Qing W, Zou M, Ke Q, Zhang L, Tang X, Nie Q, Yang Y, Hu A, Ding X, Lu L, et al. Oxidative stress-induced KLF4 activates inflammatory response through IL17RA and its downstream targets in retinal pigment epithelial cells. Free Radic Biol Med. 2020; 147:271–81. 10.1016/j.freeradbiomed.2019.12.029 31881336

[89] Crouch SP, Kozlowski R, Slater KJ, Fletcher J. The use of ATP bioluminescence as a measure of cell proliferation and cytotoxicity. J Immunol Methods. 1993; 160:81–88. 10.1016/0022-1759(93)90011-u 7680699

[90] Yan Q, Gong L, Deng M, Zhang L, Sun S, Liu J, Ma H, Yuan D, Chen PC, Hu X, Liu J, Qin J, Xiao L, et al. Sumoylation activates the transcriptional activity of pax-6, an important transcription factor for eye and brain development. Proc Natl Acad Sci USA. 2010; 107:21034–39. 10.1073/pnas.1007866107 21084637 PMC3000302

[91] Gong L, Ji WK, Hu XH, Hu WF, Tang XC, Huang ZX, Li L, Liu M, Xiang SH, Wu E, Woodward Z, Liu YZ, Nguyen QD, Li DW. Sumoylation differentially regulates Sp1 to control cell differentiation. Proc Natl Acad Sci USA. 2014; 111:5574–79. 10.1073/pnas.1315034111 24706897 PMC3992630

[92] Qin J, Chen HG, Yan Q, Deng M, Liu J, Doerge S, Ma W, Dong Z, Li DW. Protein phosphatase-2A is a target of epigallocatechin-3-gallate and modulates p53-bak apoptotic pathway. Cancer Res. 2008; 68:4150–62. 10.1158/0008-5472.CAN-08-0839 18519674

