# Supplementary material for: The transcription factor CREB acts as an important regulator mediating oxidative stress-induced apoptosis by suppressing αB-crystallin expression
Source: Aging (Albany NY). 2020 Jun 17;12(13):13594–617. doi: 10.18632/aging.103474 (PMC7377838; doi:10.18632/aging.103474)
Supplement: Supplementary Figures [file aging-12-103474-s002..pdf]

SUPPLEMENTARY FIGURES

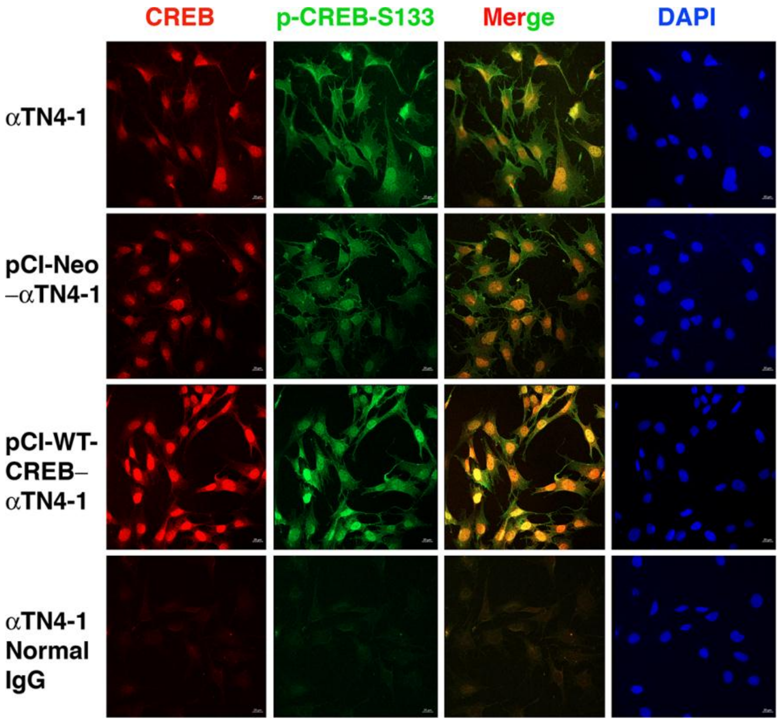

Supplementary Figure 1. Expression and localization of exogenous and endogenous CREB in αTN4-1, pCI-αTN4-1, and pCI-CREB-αTN4-1 cells. Red fluorescence represents CREB expression, green fluorescence represents p-CREB S133 expression, yellow is co-localization of CREB and p-CREB, and blue DAPI bound to DNA shows nuclear position. Note that both exogenous and endogenous CREB are highly expressed and phosphorylated in pCI-CREB-αTN4-1 cell. Both CREB and p-CREB S133 were primarily localized in the nuclei of all 3 types of cells.

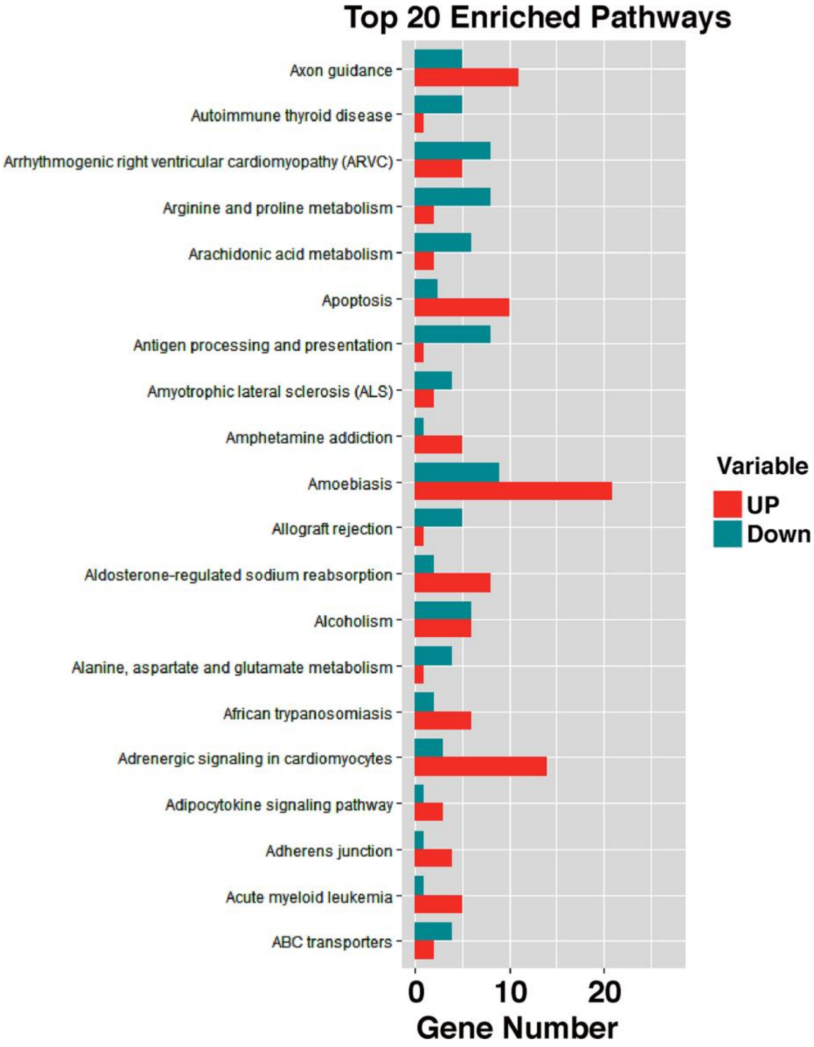

Supplementary Figure 2. Top 20 enriched pathways derived from CREB overexpression in pCI-CREB-αTN4-1 cell in comparison with pCI-αTN4-1 cell.

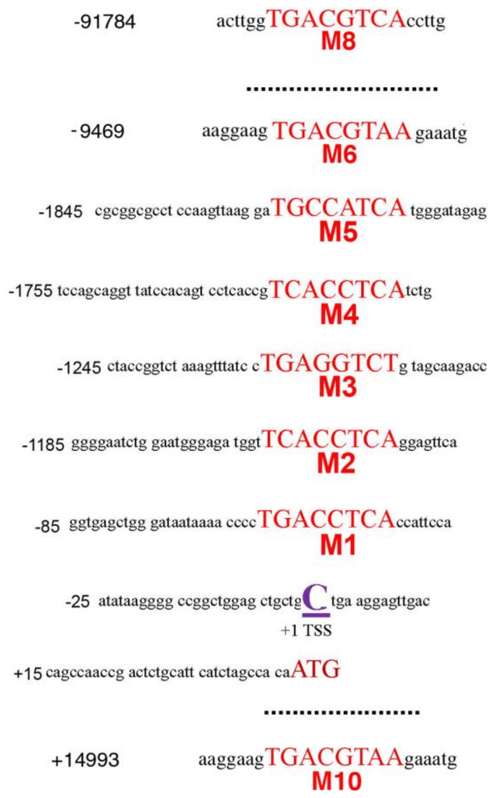

Supplementary Figure 3. Diagram to show the relative position of the full CREB binding site (TGACGTCA, M8) and its variants (M1 and M10) as well as half CREB binding sites (TGAC or GTCA or their variants) in the promoter regions, upstream or downstream enhancer regions of the αB-crystallin genomic gene. Oligos generated from M1, M8 and M10 regions were used for gel mobility shifting assays (EMSA, Figure 7A and 7B, and Supplementary Figure 4) and ChIP assays (Figure 8 and Supplementary Figure 4).

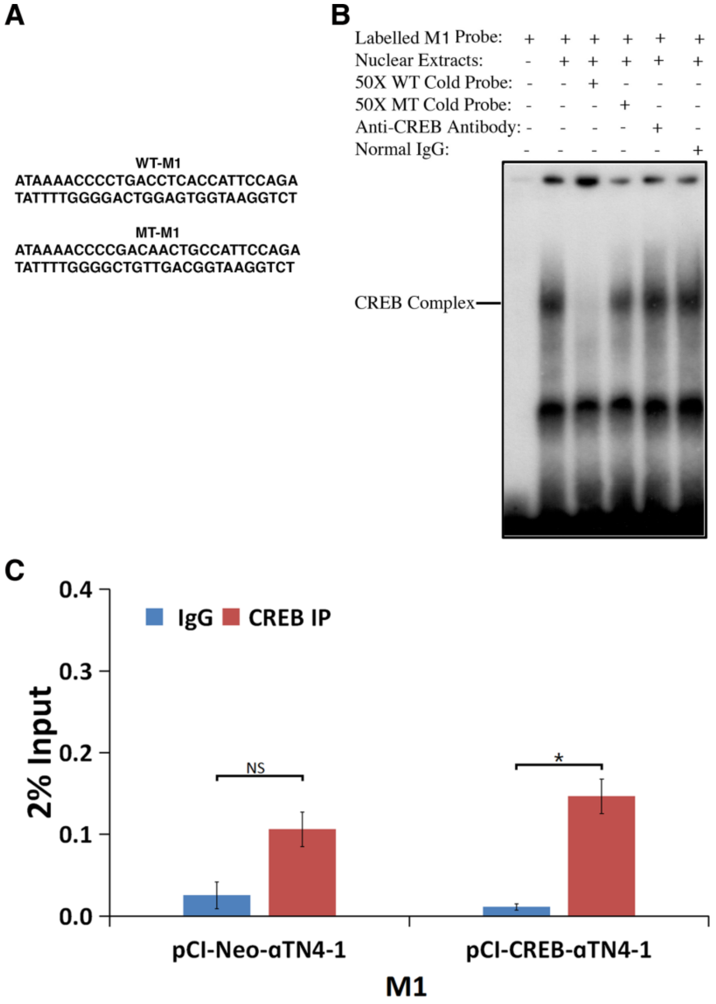

Supplementary Figure 4. Gel mobility shifting and ChIP assays to show that M1 binding site is functional. (**A**) Diagram of the two oligos containing a well-conserved CREB binding site (WT-M1, top) or mutant CREB binding site (MT-M1, bottom), which were used for gel mobility shifting assays described in **B**. (**B**) Gel mobility shifting assays. Nuclear extracts prepared from pCI-CREB-αTN4-1 cells were incubated with γ-32P-ATP-labeled oligo-nucleotide containing wild-type M1 CREB binding site (**A**) under various conditions shown in the figure. The reaction mixtures were then separated with 3.5% native PAGE. The gel was dried and exposed to X-ray film for overnight. Lane 1, gel mobility shifting assays with labeled oligo containing wild-type M1 CREB binding site but no nuclear extract from pCI-CREB-αTN4-1 cells. Lane 2, gel mobility shifting assays with labeled oligo containing wild-type M1 CREB binding site and nuclear extract from pCI-CREB-αTN4-1 cells. Lane 3, the same assay as described for lane 2 except that 50-fold of unlabeled oligo containing the wild-type M1 CREB binding site was added into the reaction. Note that the CREB complex was largely competed off by the unlabeled oligo. Lane 4, the same assay as described in lane 2 except that the unlabeled competing oligo containing a mutated CREB binding site (**A**, bottom), which could not compete off the CREB complex formed between CREB protein and the oligo containing wild-type CREB half binding site. Lane 5, the same assay as described in lane 2 except that the reaction mixtures were pre-incubated with anti-CREB antibody. Note that no supershift band was detected. Lack of the supershifting band implies that CREB may interact with another co-activator which masked the anti-CREB epitope. Lane 6, the same assay as described in lane 2 except that the reaction mixtures were pre-incubated with normal IgG. (**C**) qChIP assays to demonstrate that CREB binds to M1 region of αB-crystallin gene promoter in vivo. All experiments were repeated three times. Error bar represents standard deviation, N=3. * *$p<0.05$;* NS, statistically not significant.
