# Supplementary material for: The transcription factor CREB acts as an important regulator mediating oxidative stress-induced apoptosis by suppressing αB-crystallin expression
Source: Aging (Albany NY). 2020 Jun 17;12(13):13594–617. doi: 10.18632/aging.103474 (PMC7377838; doi:10.18632/aging.103474)
Supplement: Supplementary Table 1 [file aging-12-103474-s001..pdf]

SUPPLEMENTARY TABLE

Supplementary Table 1. Primers used in RT-qPCR analysis and ChIP-PCR assays.

Primer name	Primer direction	Primer sequences
Mouse αB-crystallin（RT-qPCR）	F	CCAGGACGAACATGGCTTCATCTC
	R	GCGACAGCAGGCTTCTCTTCAC
Mouse αB M1 (ChIP-qPCR)	F	GTCTCCATGAACTGGCGGTG
	R	GGCTGGTCAACTCCTTCAGC
Mouse αB M8 (ChIP-qPCR)	F	TCCTAGTGTCACTGAGCAGCA
	R	CGCATCAGAAGGTCTGTTCGT
Mouse αB M10 (ChIP-qPCR)	F	CTCCCGAGCAGTAGCTCCAA
	R	GGTCTCCTCCGGCTTACCTG
